# RecipeDB: a resource for exploring recipes

**DOI:** 10.1093/database/baaa077

**Published:** 2020-11-25

**Authors:** Devansh Batra, Nirav Diwan, Utkarsh Upadhyay, Jushaan Singh Kalra, Tript Sharma, Aman Kumar Sharma, Dheeraj Khanna, Jaspreet Singh Marwah, Srilakshmi Kalathil, Navjot Singh, Rudraksh Tuwani, Ganesh Bagler

**Affiliations:** Complex Systems Laboratory, Center for Computational Biology, Indraprastha Institute of Information Technology (IIIT-Delhi), New Delhi, India 110020; Complex Systems Laboratory, Center for Computational Biology, Indraprastha Institute of Information Technology (IIIT-Delhi), New Delhi, India 110020; Complex Systems Laboratory, Center for Computational Biology, Indraprastha Institute of Information Technology (IIIT-Delhi), New Delhi, India 110020; Complex Systems Laboratory, Center for Computational Biology, Indraprastha Institute of Information Technology (IIIT-Delhi), New Delhi, India 110020; Complex Systems Laboratory, Center for Computational Biology, Indraprastha Institute of Information Technology (IIIT-Delhi), New Delhi, India 110020; Complex Systems Laboratory, Center for Computational Biology, Indraprastha Institute of Information Technology (IIIT-Delhi), New Delhi, India 110020; Complex Systems Laboratory, Center for Computational Biology, Indraprastha Institute of Information Technology (IIIT-Delhi), New Delhi, India 110020; Complex Systems Laboratory, Center for Computational Biology, Indraprastha Institute of Information Technology (IIIT-Delhi), New Delhi, India 110020; Complex Systems Laboratory, Center for Computational Biology, Indraprastha Institute of Information Technology (IIIT-Delhi), New Delhi, India 110020; Complex Systems Laboratory, Center for Computational Biology, Indraprastha Institute of Information Technology (IIIT-Delhi), New Delhi, India 110020; Complex Systems Laboratory, Center for Computational Biology, Indraprastha Institute of Information Technology (IIIT-Delhi), New Delhi, India 110020; Complex Systems Laboratory, Center for Computational Biology, Indraprastha Institute of Information Technology (IIIT-Delhi), New Delhi, India 110020

## Abstract

Cooking is the act of turning nature into the culture, which has enabled the advent of the omnivorous human diet. The cultural wisdom of processing raw ingredients into delicious dishes is embodied in their cuisines. Recipes thus are the cultural capsules that encode elaborate cooking protocols for evoking sensory satiation as well as providing nourishment. As we stand on the verge of an epidemic of diet-linked disorders, it is eminently important to investigate the culinary correlates of recipes to probe their association with sensory responses as well as consequences for nutrition and health.

RecipeDB (https://cosylab.iiitd.edu.in/recipedb) is a structured compilation of recipes, ingredients and nutrition profiles interlinked with flavor profiles and health associations. The repertoire comprises of meticulous integration of 118 171 recipes from cuisines across the globe (6 continents, 26 geocultural regions and 74 countries), cooked using 268 processes (heat, cook, boil, simmer, bake, etc.), by blending over 20 262 diverse ingredients, which are further linked to their flavor molecules (FlavorDB), nutritional profiles (US Department of Agriculture) and empirical records of disease associations obtained from MEDLINE (DietRx). This resource is aimed at facilitating scientific explorations of the culinary space (recipe, ingredient, cooking processes/techniques, dietary styles, etc.) linked to taste (flavor profile) and health (nutrition and disease associations) attributes seeking for divergent applications.

**Database URL:**  https://cosylab.iiitd.edu.in/recipedb

## Introduction

A diverse, omnivorous diet is a hallmark of humans across the cultures. Cooking is the art of transforming raw ingredients into delicious dishes that humans have acquired and passed on over generations in the form of recipes. Thus the key information of processing natural ingredients into their palatable and nutritious form is encoded within the recipe instructions. Interestingly, cultures have developed distinct and diverse styles of cooking labeled broadly as a cuisine based on geocultural homogeneity. Regardless of technological interventions and changes in eating habits, the day-to-day dietary intake of people remains rooted in culture. Cooking has been argued to be central to developing large brain sizes in *Homo sapiens* (1) and critical for the gut microbiome (2). Hence, our taste preferences and health consequences are a function of the recipes.

To decode the taste, nutritional and health impacts of recipes, it is critical to build a structured repository of recipes, which are segmented and annotated into their constituent elements. Historically, the culturally transmitted recipes have had a history of being passed on orally and, lately, as written records (3). Hence, such recipes coded in natural languages are unstructured and unannotated, far from being computable.

RecipeDB was created to build a structured repository of recipes from across the globe to annotate their culinary attributes (geocultural cuisine, dietary style, cooking processes, utensils, details of their ingredients and nutritional profile), essentially making them computable. Among data resources that remotely related to the objectives of RecipeDB, FooDB focusses on the compilation of food chemicals (http://foodb.ca). FoodBase provides a resource of annotated food entities (4). Some other databases such as FlavorDB (5), BitterDB (6), SuperSweet (7) and SuperScent (8) have focused on taste and olfaction attempting to address the interaction of natural entities with human sensory machinery. Databases such as NutriChem (9) (nutritional factors), Phenol-Explorer (10, 11) (polyphenols) and DietRx have emphasized the food-nutrition-health axis (12). RecipeDB is perhaps the only resource that meaningfully integrates all relevant details of food (recipes, flavor, health and nutrition).

**Figure 1.**
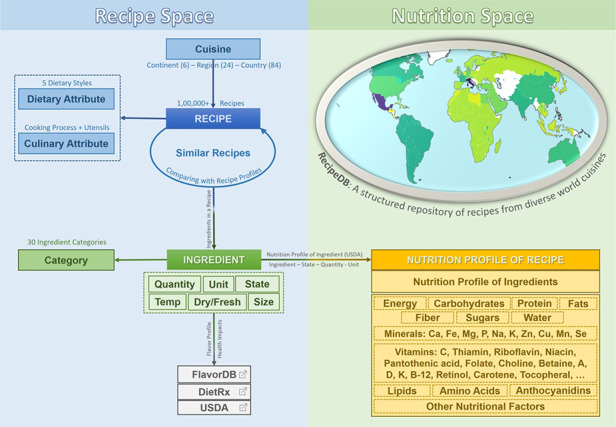
RecipeDB integrates the elements and attributes (Recipe Space) with the USDA nutritional profiles (Nutrition Space) and also links them to flavor and health attributes. The resource provides comprehensive coverage of worldwide recipes from across 26 world regions and 74 countries.

RecipeDB presents a structured, annotated compilation of over 118 171 recipes from across the globe to probe their culinary, nutritional, flavor and health correlates (Figure 1). By dissecting the recipes into their culinary elements using state-of-the-art algorithms, it provides a searchable database of recipes. Beyond the geocultural coordinates of recipes, it also provides their dietary styles and characterizes them based on the processes and utensils used for cooking. It accounts for the temporal sequence of processing and provides a structured breakdown of every ingredient phrase into the name, quantity, unit, state and other relevant attributes. Importantly, these are integrated with the US Department of Agriculture (USDA) nutrition data. Further, it links ingredients to FlavorDB (5), the most comprehensive repertoire of flavor compounds from natural ingredients and DietRx (13), a database that compiles health impacts of food ingredients from MEDLINE using a text-mining protocol.

## Database overview

RecipeDB is a resource with extensive coverage of 118 171 recipes, which are composed of 23 548 ingredients (Figure 1). The recipes have been classified into cuisines represented by 26 geocultural regions that span 6 continents and containing 74 countries (a detailed description of the protocol followed is available in ‘Geocultural Mapping of Recipes’ section) (14). The ingredients have been grouped into 29 categories via exclusive mappings (5, 14). Recipes are also labeled with 5 dietary styles and annotated for 268 cooking processes and 69 utensils that are used in these recipes. The protocol followed for dietary style labeling is mentioned in detail in the ‘Dietary Style Annotation’ section.

RecipeDB offers a user-friendly interface for querying and browsing recipes and related details via their geocultural mappings, dietary style, estimated nutrition and constituent ingredients. Interactive data visualizations and interlinked search options are provided to retrieve relevant information. Apart from searching via textual query, RecipeDB also provides a ‘Visual Search’ for interactive browsing through the ingredients and ingredient categories to access recipe details. For any recipe, the resource also facilitates lookup for similar recipes within the database. Thus, by blending recipes with their estimated nutrition profile with a dynamic interface and visualizations, RecipeDB provides a wide spectrum of information, facilitating insights into the culinary attributes of recipes.

## Data compilation

To begin with, recipes were aggregated from the following sources: GeniusKitchen (http://www.geniuskitchen.com; now renamed as http://www.food.com) and AllRecipes (http://allrecipes.com). While we reviewed a large number of recipe repositories as a potential source of data beyond these two (Tarladalal (http://www.tarladalal.com), The Spruce Eats (https://www.thespruceeats.com), Epicurious (https://www.epicurious.com), Food Network (https://www.foodnetwork.com) and taste.com.au (https://www.taste.com.au)), we chose these based on the uniformity in structure, availability of geocultural mapping and number of recipes. Each recipe was divided into two parts, the ingredients section and the cooking instructions section to extract relevant information out of them.

### Ingredients data

To construct the dataset, we required information of each recipe from the dataset in a structured format. The ingredients section includes information regarding the ingredients used in the recipe and their corresponding attributes. We identified seven attributes, which provide relevant nutritional information of a recipe. The attributes include the following: Name—Name of ingredient, e.g. salt, pepper; State—Processing state of ingredient, e.g. ground, thawed; Unit—Measuring unit of ingredient, e.g. gram, cup; Quantity—Quantity associated with unit (s), e.g. 1 teaspoon, 10 g; Size—Portion sizes mentioned, e.g. small, large; Temperature—Temperature applied prior to cooking, e.g.—hot, cold; Dry/fresh—Is the ingredient dry or fresh?

While extracting ingredients and their corresponding attributes, we primarily faced following three challenges. Unknown Attributes: There is no known list of attributes, which we could use to extract the required information. Moreover, our database of recipes is immensely varied, which further complicates the task. Identification of Attributes: Homograph attributes are common in recipes. For example, ‘clove’ may refer to the ingredient itself or a unit of measurement. Variation in Lexical Structure of Ingredient Phrases: A phrase in the ingredients section may be written in several different ways. The lexical structure of the sentence may be as simple as ‘1 cup sugar’ or a more complex structure such as ‘(8 ounce) package cream cheese, softened’.

To handle these challenges, a robust pipeline is required to extract the desired ingredient attributes from a recipe. For the ingredients section specifically, we utilized a part of the pipeline introduced in Diwan *et al.* (15). The pipeline comprises of the following seven steps (1). Dataset of ingredients: First, we constructed a dataset of approximately 10 million ingredient phrases from the database of 118 000 recipes from AllRecipes.com and FOOD.com (2). Part-of-Speech (POS) Tagging: Next we tagged each word in every ingredient phrase by a tagger. The tags were POS tags such as noun, proposition etc. All the ingredient phrases were tagged by 36 unique tags using the Stanford POS Twitter model (16) since an ingredient phrase resembles a short sentence (a tweet) than a normal sentence (3). Representation Vector: Next to represent an ingredient phrase we use a Bag of Words (BOW) model. An ingredient phrase was represented by the frequency of the POS tags in the phrase. For example, a phrase such as ‘2 cans stewed tomatoes’ was tagged as ‘2_CD cans_NNS stewed_JJ tomatoes_NNS’ where the tags refer to CD—Cardinal Number, NNS—Noun Plural and JJ—Adjective. The corresponding frequency of the tags forms the vector. Thus, we obtain a 1 × 36 representation vector for each ingredient phrase (4). Clustering: Next, we attempt to identify unique representation vectors we could use to increase the diversity of our subsequent NER model. To do this, we cluster the representation vectors using Spherical k-means clustering algorithm (17) and identify 27 clusters (via elbow method), which represent unique representation vectors (5). Dataset for NER model: For the AllRecipes corpus, we selected 1% ingredient phrases from each cluster to form a training set of 1500 ingredient phrases. Similarly, 0.33% ingredient phrases from each cluster were taken to form a test set of 500 ingredient phrases. Similarly, for the FOOD.com corpus, we selected 0.5% ingredient phrases from each cluster to form a training set of 5000 ingredient phrases; 0.15% ingredient phrases from each cluster were taken to form a test set of 1500 ingredient phrases. A smaller percentage were used in the case of FOOD.com corpus due to its size (102 000 Recipes). In summary, we created three datasets with training and testing set division for each: AllRecipes (Training Set: 1470, Testing Set: 483), FOOD.com (Training Set: 5142, Testing Set: 1750) and both datasets together (Training Set: 6612, Testing Set: 2188) (6). Tagging Dataset: Next we manually tagged each word in the ingredient phase in the training and test set using the NAME, STATE, SIZE, QUANTITY, UNIT, TEMP and DRY/FRESH, based on the attributes mentioned above (7). Named Entity Recognition (NER) Model—Next, we utilize the Stanford NER (18) to train three NER models using the abovementioned datasets.

We tested all the three models on each of the three testing sets and compared the macro-F1 score (F1 score averaged across all classes) across all the models (Table 1). We observe that the models expectedly perform the best when tested upon their respective parent datasets. The model trained upon data from both AllRecipes and FOOD.com dataset performs exceedingly well (F1 score ≥ 0.95) on all three test sets. Beyond the NER model proposed in this article (15), many efforts have been made toward food information extraction recently (19).

**Table 1.** Comparison of macro F1 scores across all models for different testing dataset used

Testing dataset	Model trained upon		
	AllRecipes	FOOD.com	Both
AllRecipes	0.9682	0.9317	0.9707
FOOD.com	0.8672	0.9519	0.9498
Both	0.8972	0.9472	0.9611

We also compared the class-wise performance of the best model (Table 2). The classes which were the most easily tagged were QUANTITY (F1 score = 0.9928) and UNIT (F1 score = 0.9824). The classes that are hardest to classify were NAME (F1 score = 0.9251) and TEMP (F1 score = 0.9286). All experiments are 5-fold cross validated. Following is the github link for the manually curated train and test data: https://github.com/cosylabiiit/Recipedb-companion-data.

**Table 2.** Performance metrics for different classes

Entity	Precision	Recall	F1 score
NAME	0.9300	0.9203	0.9251
STATE	0.9447	0.9632	0.9539
UNIT	0.9869	0.9781	0.9824
QUANTITY	0.9959	0.9896	0.9928
TEMP	0.9512	0.9070	0.9286
SIZE	0.9596	0.9500	0.9548
Dry/Fresh	0.9667	0.9732	0.9699
Total	0.9656	0.9610	0.9633

### Cooking instructions data

Similarly, in the instructions section, an NER model was trained to identify cooking processes, ingredients and utensils using the Stanford NER (18). The recipes with the longest instructions section from 40 different cuisines were extracted to further annotate 268 processes/techniques, ingredients and 69 utensils. The Stanford NER tagger (18) was trained upon this corpus, and the resulting model was used to get inferences for all the recipes in the database. Out of 2086 sentences (extracted from 120 longest recipes from across all cuisines) that were manually annotated, 1586 were randomly chosen for training and the rest (500) were used for testing. The models presented with F1 score of 0.88 (Precision: 0.92 and Recall: 0.85) and 0.90 (Precision: 0.94 and Recall: 0.86), respectively, for processes and utensils, respectively. The cooking processes and utensil names thus obtained were filtered manually to create a dictionary of processes and utensils, which appear above a threshold frequency (47 and 10, respectively). Supplementary Figure S1 and Supplementary Figure S2 present a graphical overview of the representation of cooking processes/techniques and utensils recipes across the world regions, respectively. The recipe instructions were chronologically split into three approximately equal parts (Early Stage, Middle Stage and Late Stage).

### Geocultural mapping of recipes

Every recipe was mapped to its geocultural correlate at three levels of hierarchy: Continent, Region and Sub-region (country). The protocol followed for geocultural mapping of recipes was consistent with the one used by Singh and Bagler in their data-driven investigation of recipes (14). Continent and country mapping for all the recipes were acquired from the data sources. Region level mapping of recipes was done primarily based on their culinary/cultural similarities rather than their geological relationship. For example, although Egypt geographically is a part of Africa, it was classified as a member of the Middle Eastern region because of its culinary/cultural similarities with the region. Furthermore, countries with a large number of recipes such as Canada, Mexico, Italy and the US formed their own sub-regions. For countries with fewer recipes, broader sub-regions were created, such as ‘Rest Caribbean’. A similar protocol was followed for the African continent in which due to the lack of specific mapping to a country of origin, such recipes were bundled under the sub-region ‘Rest Africa’. Supplementary Figure S3 presents a graphical overview of the nature of distribution of recipes across the world regions. Supplementary Table S1 and Supplementary Table S2 present the statistics and geocultural cuisine mappings.

### Ingredients annotations

Each of the ingredients was manually mapped to one of the 29 categories (Additive, Additive-Salt, Additive-Sugar, Additive-Vinegar, Additive-Yeast, Bakery, Beverage, Beverage-Alcoholic, Beverage-Caffeinated, Cereal, Condiment, Condiment-Sauce, Dairy, Dish, Essential Oil, Fish, Flower, Fruit, Fungi, Herb, Legume, Maize, Meat, Nuts and Seeds, Plant, Plant Derivative, Seafood, Spice and Vegetable) exclusively. The list of ingredient categories available from FlavorDB was used as the baseline (5). Each ingredient was also assigned with a ‘generic ingredient name’ manually and linked to its Wikipedia page. For example, all the different variations of chili peppers were aggregated with the generic name of ‘chili pepper’ and were linked to the Wikipedia page for ‘chili pepper’. The generic ingredients names were further mapped to their corresponding flavor profile from FlavorDB (5) and disease association profile from DietRx (13), when available. Supplementary Figure S4 and Supplementary Figure S5 present a graphical overview of the category composition of the recipes across the world regions and representation of ingredient of each category, respectively.

### Dietary style annotation

Each recipe was tagged with one of the following commonly accepted and widely practiced dietary styles: Vegan, Pescetarian, Lacto-Vegetarian, Ovo-Vegetarian and Ovo-Lacto-Vegetarian. Rules were created for designating a dietary style based on the ingredient category and/or ingredient(s) used in a recipe. For each dietary style, the rules were formed to specify categories of ingredients that were restricted and the ones mandatory. For a recipe to be associated with a dietary style, it needed to have no ingredient belonging to the dietary style’s list of restricted categories while having at least one or more ingredients belonging to each of the dietary style’s list of mandatory categories. As a precaution, any recipe containing an ingredient under the category ‘bakery’ was assumed to have eggs in it. Also, a recipe was not mapped to any dietary style if it had an ingredient(s) of the ‘dish’ category. Following were the rules used: Vegan (Exclude: meat, eggs, dairy, fish, seafood, dish); Pescetarian (Include: fish or seafood and Exclude: meat, dairy, dish); Lacto-Vegetarian (Include: dairy and Exclude: meat, eggs, fish, seafood, dish); Ovo-Vegetarian (Include: egg and Exclude meat, fish, seafood, dairy, dish); and Ovo-Lacto Vegetarian (Include: egg and dairy and Exclude: meat, fish, seafood, dish).

### Estimating nutritional values of recipes

Nutrition value of the recipes was assumed to be the sum total of the nutrition value of its constituent ingredients. For this calculation, each ingredient in a recipe was mapped to a corresponding NDB ID in the Standard Reference Legacy Release database (https://fdc.nal.usda.gov) (whenever possible) by matching the ingredient information (name and state) with the ‘long description of food’ using the ‘Jaccard similarity’ algorithm (20). Thereafter, the nutritional values of the ingredients as used in the recipe were estimated by matching the quantity and unit of the ingredient in the recipe to one of the corresponding units present in the USDA dataset of the matched ingredient. In case of not finding a match, ‘measurement conversion tables’ were used where numerical relationships between different units were defined. If this NER process could not detect any unit for an ingredient, the most frequently used unit was used. Supplementary Figure S6 presents a graphical overview of the nature of distribution of nutritional content of recipes across the world regions.

## Database architecture and web interface

**Figure 2.**
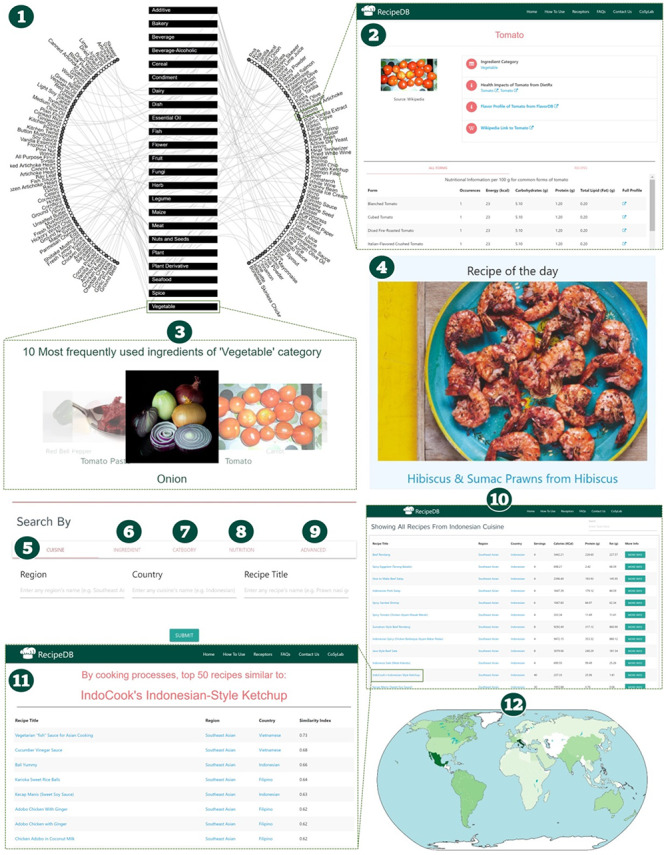
Schematic of RecipeDB user interface highlighting features for searching and navigation of data (1). Recipe Network (2), Ingredient Profile (3), Category Profile (4), Ingredient of the day, (5)–(9) Search by Cuisine, Ingredient, Category, Nutrition and Advanced Search, (10) Results page for recipes search, (11) Similar recipes page and (12) RecipeDB Statistics (Recipes, Categories, Ingredients, Nutrition, Processes and Utensils).

RecipeDB facilitates easy comprehension and navigation of complex interrelations among the cuisines, recipes, ingredients and their categories (Figure 2). Interactive data visualizations and a wide variety of user-friendly searches provide quick access to the desired information. The following utilities and applications in RecipeDB enable explorations of recipes and ingredients to get insights into the culinary world.

### Visual search

Recipe Network visualizes the relationship between ingredients and ingredient categories to provide an interactive way of exploring RecipeDB. The ingredient categories are represented as boxes in the middle of the graph (all additives were merged into one category and beverage was merged with beverage-caffeinated), while the ingredients are laid out along the circumference to make it easier to observe the relationships among entities. To address the dense pattern of relationships, the six most frequently used ingredients from each category are depicted. Hovering over an entity shows its association with other entities. One can navigate the network by either clicking on the ingredient category or one of the ingredients. The visual search lands either on the category profile page or on the ingredient profile page. Thus, visual search enables open-ended exploration of RecipeDB. The Flavor Network was implemented with the D3.js JavaScript library (https://d3js.org).

### Recipe of the day

‘Recipe of the Day’, prominently featured on the landing page, initiates the beginner user by presenting a random recipe from the database. This feature offers a peek into the RecipeDB, from where the user can start exploring the resource.

### Recipe search

‘Recipe Search’ forms a key querying mechanism that enables search via the following features: cuisine, ingredient, ingredient category and nutrition. It facilitates querying on the basis of a host of features, including cuisine (continent, geocultural region and country), ingredients used/not used and nutritional profile (calories, carbohydrates, protein and fat). Structurally similar recipes can be found using the ‘Search Similar in RecipeDB’ button provided on the recipe profile pages. Recipe search yields matching recipes with detailed profiles. RecipeDB also facilitates browsing all 118 171 recipes by doing a null search (no constraints; all query fields empty).

### Advanced search

Advanced search provides an option for refined search by querying RecipeDB data on the basis of a combination of all the separate search features provided in Recipe Search. Beyond the options provided in the Search panels, advanced search enables querying on 268 cooking process/techniques and 69 utensils.

### Recipe profile

Every recipe is presented with a detailed profile with the following sections: Estimated nutritional profile, Recipe overview, Ingredients, Processes, Utensils and Detailed recipe instructions. Beyond the nutritional profile at the level of the recipe and individual ingredients, the profile provides an overview (cuisine mapping, preparation time when available and out-link to the source). Importantly, it provides a temporal sequence of cooking processes/techniques and utensils used in the recipe.

### Similar recipes

Having obtained the recipe structure in its basic constituent elements, one can now seek similar recipes by way of ingredient category composition or the cooking processes/techniques. Using ‘Similar Recipes by Category Composition’ and ‘Similar Recipes by Processes’ button on the recipe profile page, one can obtain recipes that align with the present recipe in the multidimensional space of cooking techniques or ingredients category. Thus, RecipeDB enables searching for ‘similar recipes’ in the vast space of recipes enabling discovery of new recipes and patterns.

Each recipe was translated to one-hot encoded vector of dimension (1 × 20 280) and (1 × 268) to create the recipe-ingredient and recipe-process matrices, respectively. Cosine distances between the vectors were calculated and top 50 similar recipes were presented in response to a query. Considering the fact that each recipe consists of around 10 ingredients and 12 processes on an average, both matrices are highly sparse. Therefore, the matrices were converted to condensed sparse row matrices to improve the efficiency of the computation. The cosine distance ranges from 0 to 1 with the former representing to the highest similarity and latter pointing to the least similarity. Accordingly, the similarity index was defined as (1—cosine distance).

### Ingredient profile

For each generic ingredient (1636) RecipeDB presents its profile page with details of its category, Wikipedia link and, whenever available, link-out to health impacts (Die-tRx (13); https://cosylab.iiitd.edu.in/dietrx) and their flavor profile (FlavorDB (5); http://cosylab.iiitd.edu.in/flavordb). One can explore these in detail to probe the health and flavor correlates of the ingredient, respectively.

### Ingredient category profile

Each of the ingredient categories is presented with its detailed profile page. The page presents a visual gallery of the top 10 most frequently used ingredients of that category and also link-outs to the top 20 popular ingredients. It also presents the top 20 recipes that have dominant representation (number of ingredients) of ingredients from the chosen category.

### Taste and odor receptors

The perception of food happens primarily via sensory mechanisms of taste and odor. RecipeDB provides an exhaustive, structured list of taste and odor receptors. These were curated from Uniprot (21) by using the keywords ‘Bitter/Sour/Sweet/Umami/Odor and Receptor’ followed by manual curation. For example, for the keyword ‘bitter receptor’, the list of receptors rendered was manually verified and was filtered to remove false positives.

### Webserver tech stack

RecipeDB has been designed as a Relational Database using SQLite (https://www.sqlite.org/index.html). The webserver has been built using the Python web development framework, Flask (flask.pocoo.org). Flask has agnostic Object Relational Mapper (ORM) for querying the database using any of the ORMs, thus optimizing queries and making it easier to perform complex queries, apart from reducing the development period. The front end has been built using HTML, CSS and JavaScript. The jQuery (https://jquery.com/), MaterializeCSS (https://materializecss.com/), D3.js (https://d3js.org/) and plotly (https://plot.ly/) were used to add to the functionality of RecipeDB. An NGINX (https://www.nginx.com/) HTTP Server has been used to route requests to the Flask app and to enable data compression for faster page load times. The site is best viewed in the latest versions of Google Chrome, Firefox, Opera, Internet Explorer and Microsoft Edge.

## Use cases

Below we provide a few case studies illustrating the utility of RecipeDB for various applications.

### Searching recipes by cuisine

One may search for recipes by the cuisine at the level of ‘region’ of ‘country’. For example, one may search by the ‘Southeastern Asian’ region followed by ‘Indonesian’ as Country or directly by the latter. Each field is powered with a single letter autosuggest to enable an uninitiated user. The result page returns the list of all Indonesian recipes (18 pages; 20 recipes per page) present in RecipeDB along with their names, estimated macronutrients, as well as link-outs to the recipe pages and detailed estimated nutritional profiles. Clicking on the recipe name would yield a page with structural details of the recipe, and clicking on the ‘More Info’ tab would open a pop-out a page with estimated nutritional profile for macro- as well as micro-nutrients as available in USDA. One can move across the results using Previous, Next links or by jumping to any page number from the panel presented at the bottom and also search the page using the search box provided at the top.

### The estimated nutritional profile of a recipe

Using the protocol described in the ‘Data Compilation’ section (‘Estimating Nutritional Values of Recipes’), the nutritional profile of each recipe has been estimated. For example, the ‘Indonesian Pork Satay’ (4 servings) has estimated calories of 1647.39 KCal and protein and fat of 179.12 and 84.59 g, respectively. Clicking on the ‘More Info’ button provides a full estimated nutritional profile of micro- as well as macro-nutrients. A nutritional profile breakdown into its individual elements/ingredients is also provided.

### Recipe profile and similar recipes

From its recipe profile, we observe that ‘Indonesian Pork Satay’ involves, among other things, pouring, mixing in early-stage, marinating in mid-stage and boiling in late stage. The recipe mentions processor and saucepan as utensils being used. The ‘Instructions’ section provides detailed recipe instructions as obtained from the source.

Exploring for similar recipes of ‘Indonesian Pork Satay’, the ‘Spicy Indonesian Pork Satay (Or Chicken)’ comes as the closest recipe with a similarity index of 0.64 followed by ‘Grilled Chicken Adobo’ (0.59) using cooking techniques as the measure of similarity. By category composition, the closest recipes are ‘Spicy Pork Baked Ziti With Ragu’ (0.92) and ‘Delicious Bolognese Meat Sauce’ (0.92).

### Exploring flavor molecules and health impacts

RecipeDB facilitates exploring the molecular basis of the flavor and empirically reported health impacts of ingredients of its recipes via out-links to FlavorDB (5) and DietRx (13). For example, in the case of ‘Indonesian Pork Satay’, one of its ingredients ‘garlic’ has been reported to have significant beneficial effects against Cancer (Neoplasms; Disease ID: D009369). The flavor profile of garlic presents detailed information of garlic, its natural source (Allium), its flavor molecules and facilitates finding flavor pairing of garlic with other ingredients. FlavorDB facilitates exploration of the flavor profile of the ingredients as well as finding ways for tweaking recipes based on the similarity/complementarity of the flavors.

### Searching recipes by ingredient or its category

RecipeDB enables searching for recipes by the presence or absence of an ingredient or ingredient category. For example, searching for all recipes ‘having spinach’ and ‘not having onion’ in them returns 148 pages of results. Similarly, searching for all recipes ‘having dairy’ and ‘not having spice’ in them returns 3198 pages of results. Such queries enable one to seek for recipes across the global repository in a structured manner.

### Searching recipes by macronutrients

Searching for recipes having the desired quantity of macronutrients (Energy, Carbohydrates, Proteins and Fats) is also facilitated by RecipeDB. With default values set around one standard deviation around the mean of all recipes, the interface allows changing the search range.

### Complex queries with advanced search

The advanced search enables creating nuanced queries using the individual query elements (cuisine, recipe title, ingredient used/not used, cooking processes and utensils used and macronutrients). For example, one may search for all Indonesian recipes ‘having spinach’, ‘not having onion’ and using cooking process ‘fry’ returns two recipes: ‘Indonesian Sweet and Sour Tofu With Vegetables’ and ‘Nasi Goreng’.

## Discussion

Food is a complex subject interwoven with traditional cooking practices (recipes), flavor, nutrition and health. The ‘Complex Systems Laboratory’ at IIIT-Delhi has been putting together the data-driven perspective of these jig-saw pieces of the food puzzle in the form of a ‘Computational Gastronomy’ framework including FlavorDB (5), DietRx (13), taste prediction algorithms (BitterSweet machine learning models (22)) and a body of research (23–25) that encompasses food pairing, evolution of cuisines and the basis for the dietary use of herbs and spices.

RecipeDB provides one of the most structured repositories of worldwide recipes to integrate cultural, culinary and nutritional aspects with those of flavor and health impacts. While a plethora of (web based as well as printed) recipe resources are available, which archive recipes in a human-readable format, none looks at recipes from a data-centric perspective intended to capture recipe elements for making recipes computable. By creating a curated and structured culinary knowledgebase, RecipeDB fills in this gap and enables open-ended explorations.

However, despite an impressive performance of the algorithms to extract relevant information from the recipe instructions, RecipeDB has much scope to improve in gathering the following elements: unit and quantity of an ingredient and the exact process/technique being applied to the ingredient. The former is critical for the estimation of the nutritional profile of the ingredient and, therefore, of the recipe. When encountered with a ‘null unit’, the present algorithm uses a heuristic for assigning the most frequently used unit for the ingredient, which may, at times, be incorrect. The latter is missing in RecipeDB at the moment and is a key factor determining the nutritional profile of the recipes.

Other than improving the quantity of the data, there is much scope for improving the quality of the recipes data. The present data compilation strategy relies on the classification of the recipes done by crowd-sourced resources (AllRecipes and Genius Kitchen), which may not be entirely accurate. Compilation of generic traditional recipes is one among the key future directions. Many of the recipes compiled by us could, in fact, be better classified as ‘International Standard Cuisine’. Correspondingly, the estimated nutritional values could change due to use of nonstandard ingredients. Our strategy uses USDA data for nutritional mapping. Going further, it may be more relevant to use national nutritional databases.

The data gathered from RecipeDB is a potent source for the analysis of ‘identity’ of cuisine in measurable parameters, similarity among cuisines, quintessential patterns in cuisines, among others, apart from being an excellent resource for asking queries. Thus, RecipeDB provides a quantified resource of the world’s culinary heritage through a structured repository of recipes.

## Supplementary Material

baaa077_Supp

## Availability

RecipeDB is available at https://cosylab.iiitd.edu.in/recipedb under the Creative Commons Attribution-NonCommercial-ShareAlike 3.0 Unported License.
