# Supplementary material for: RecipeDB: a resource for exploring recipes
Source: Database (Oxford). 2020 Nov 25;2020:baaa077. doi: 10.1093/database/baaa077 (PMC7687679; doi:10.1093/database/baaa077)
Supplement: baaa077_Supp [file baaa077_supp.zip › baaa077_RecipeDB-Manuscript_v3p0_REVISION_16July2020_SUPPLE.docx]

S**UPPLEMENTARY MATERIAL**

| **Region** | **Number of Recipes** |
| --- | --- |
| Australian | 5823 |
| Belgian | 1060 |
| Canadian | 6700 |
| Caribbean | 3026 |
| Central American | 460 |
| Chinese and Mongolian | 5896 |
| Deutschland | 4323 |
| Eastern European | 2503 |
| French | 6381 |
| Greek | 4185 |
| Indian Subcontinent | 6464 |
| Irish | 2532 |
| Italian | 16582 |
| Japanese | 2041 |
| Korean | 668 |
| Mexican | 14463 |
| Middle Eastern | 3905 |
| Northern Africa | 1611 |
| Rest Africa | 2740 |
| Scandinavian | 2811 |
| South American | 7176 |
| Southeast Asian | 1940 |
| Spanish and Portuguese | 2844 |
| Thai | 2605 |
| UK | 4401 |
| US | 5031 |
| **Total** | **118171** |

**Supplementary Table S1**: Number of recipes across cuisines at the regions-level.

| **Continent** | **Region** | **Sub-region (Country)** |
| --- | --- | --- |
| African | Middle Eastern | Egyptian |
| African | Northern Africa | Libyan |
| African | Northern Africa | Moroccan |
| African | Rest Africa | Angolan |
| African | Rest Africa | Ethiopian |
| African | Rest Africa | Namibian |
| African | Rest Africa | Nigerian |
| African | Rest Africa | Somalian |
| African | Rest Africa | Sudanese |
| Asian | Chinese and Mongolian | Chinese |
| Asian | Chinese and Mongolian | Mongolian |
| Asian | Indian Subcontinent | Bangladeshi |
| Asian | Indian Subcontinent | Indian |
| Asian | Indian Subcontinent | Nepalese |
| Asian | Indian Subcontinent | Pakistani |
| Asian | Japanese | Japanese |
| Asian | Korean | Korean |
| Asian | Middle Eastern | Iraqi |
| Asian | Middle Eastern | Israeli |
| Asian | Middle Eastern | Laotian |
| Asian | Middle Eastern | Lebanese |
| Asian | Middle Eastern | Palestinian |
| Asian | Middle Eastern | Rest Middle Eastern |
| Asian | Middle Eastern | Saudi Arabian |
| Asian | Middle Eastern | Turkish |
| Asian | Southeast Asian | Cambodian |
| Asian | Southeast Asian | Filipino |
| Asian | Southeast Asian | Indonesian |
| Asian | Southeast Asian | Malaysian |
| Asian | Southeast Asian | Vietnamese |
| Asian | Thai | Thai |
| Australasian | Australian | Australian |
| Australasian | Australian | New Zealander |
| European | Belgian | Belgian |
| European | Belgian | Dutch |
| European | Deutschland | Austrian |
| European | Deutschland | German |
| European | Deutschland | Swiss |
| European | Eastern European | Czech |
| European | Eastern European | Hungarian |
| European | Eastern European | Polish |
| European | Eastern European | Rest Eastern European |
| European | Eastern European | Russian |
| European | French | French |
| European | Greek | Greek |
| European | Irish | Irish |
| European | Italian | Italian |
| European | Scandinavian | Danish |
| European | Scandinavian | Finnish |
| European | Scandinavian | Icelandic |
| European | Scandinavian | Norwegian |
| European | Scandinavian | Swedish |
| European | Spanish and Portuguese | Portuguese |
| European | Spanish and Portuguese | Spanish |
| European | UK | English |
| European | UK | Scottish |
| European | UK | UK |
| European | UK | Welsh |
| Latin American | Caribbean | Cuban |
| Latin American | Caribbean | Jamaican |
| Latin American | Caribbean | Puerto Rican |
| Latin American | Caribbean | Rest Caribbean |
| Latin American | Central American | Costa Rican |
| Latin American | Central American | Guatemalan |
| Latin American | Central American | Honduran |
| Latin American | Mexican | Mexican |
| Latin American | South American | Argentine |
| Latin American | South American | Brazilian |
| Latin American | South American | Chilean |
| Latin American | South American | Colombian |
| Latin American | South American | Ecuadorean |
| Latin American | South American | Peruvian |
| Latin American | South American | Venezuelan |
| North American | Canadian | Canadian |
| North American | US | US |

**Supplementary Table S2**: The geo-cultural mappings of the recipes at the level of continent, region and sub-region (country).

**
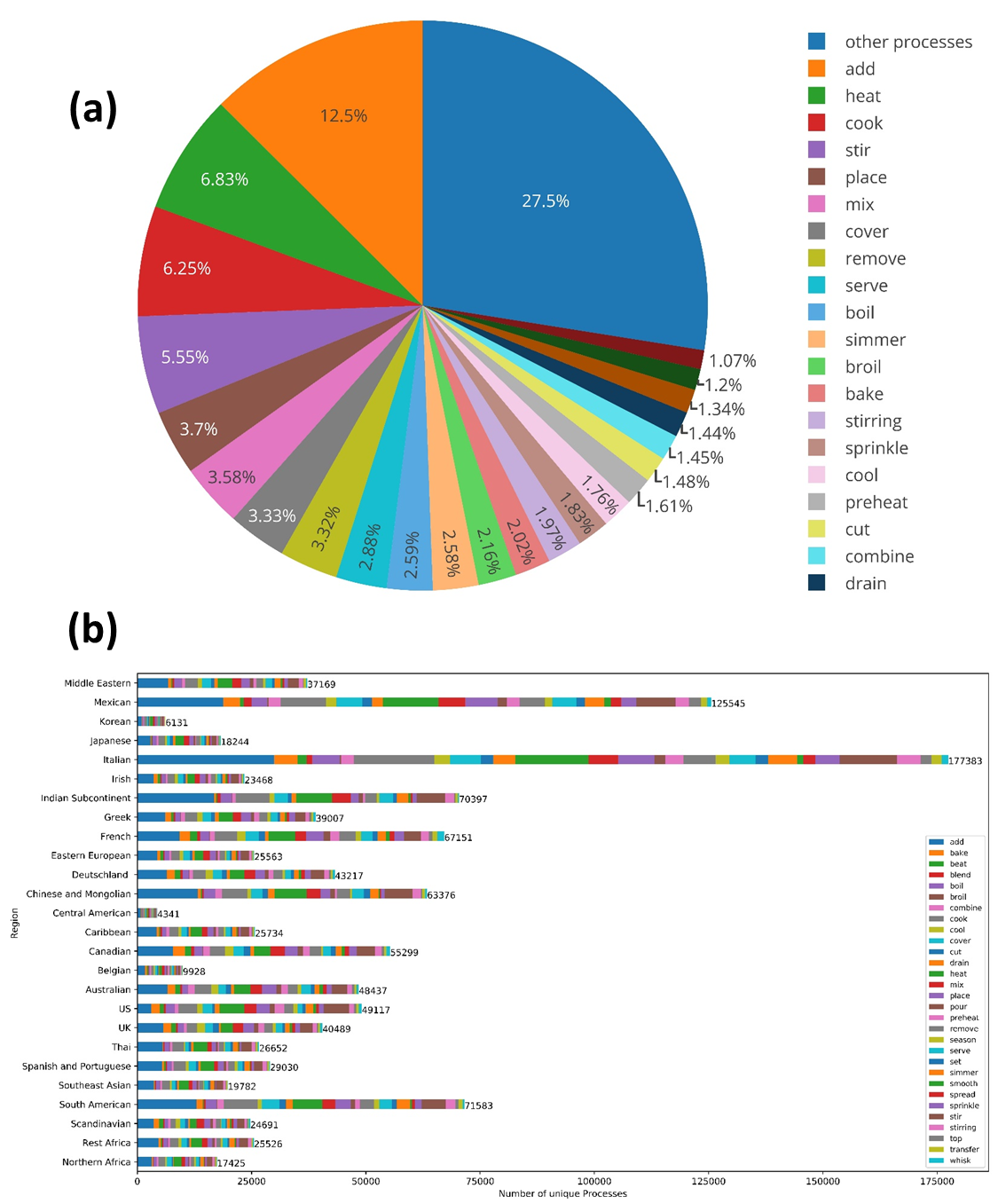
**

**Supplementary Figure S1**: (a) The pie chart shows the representation of cooking processes/techniques (268) that are used across the worldwide recipes. Beyond the major techniques that are predominantly used in cooking, the rest are bundled into ‘other processes’ for the ease of visualization. (b) The bar chart shows the statistics for cuisines at the region-level.

**
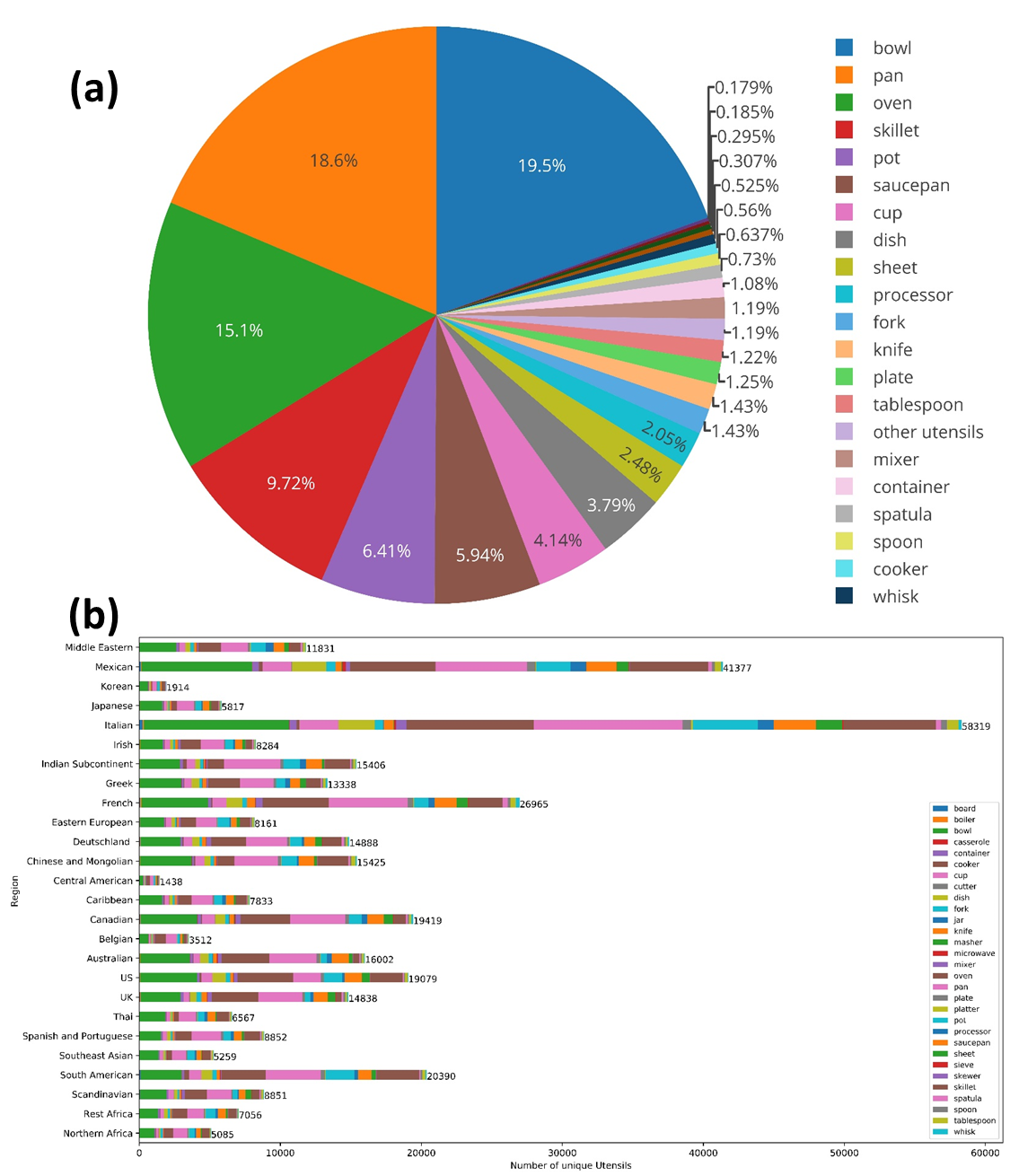
**

**Supplementary Figure S2**: (a) The pie chart shows the representation of cooking utensils (69) that are used across the worldwide recipes. Beyond the major utensils that are predominantly used in cooking, the rest are bundled into ‘other utensils’ for the ease of visualization. (b) The bar chart shows the statistics for cuisines at the region-level.


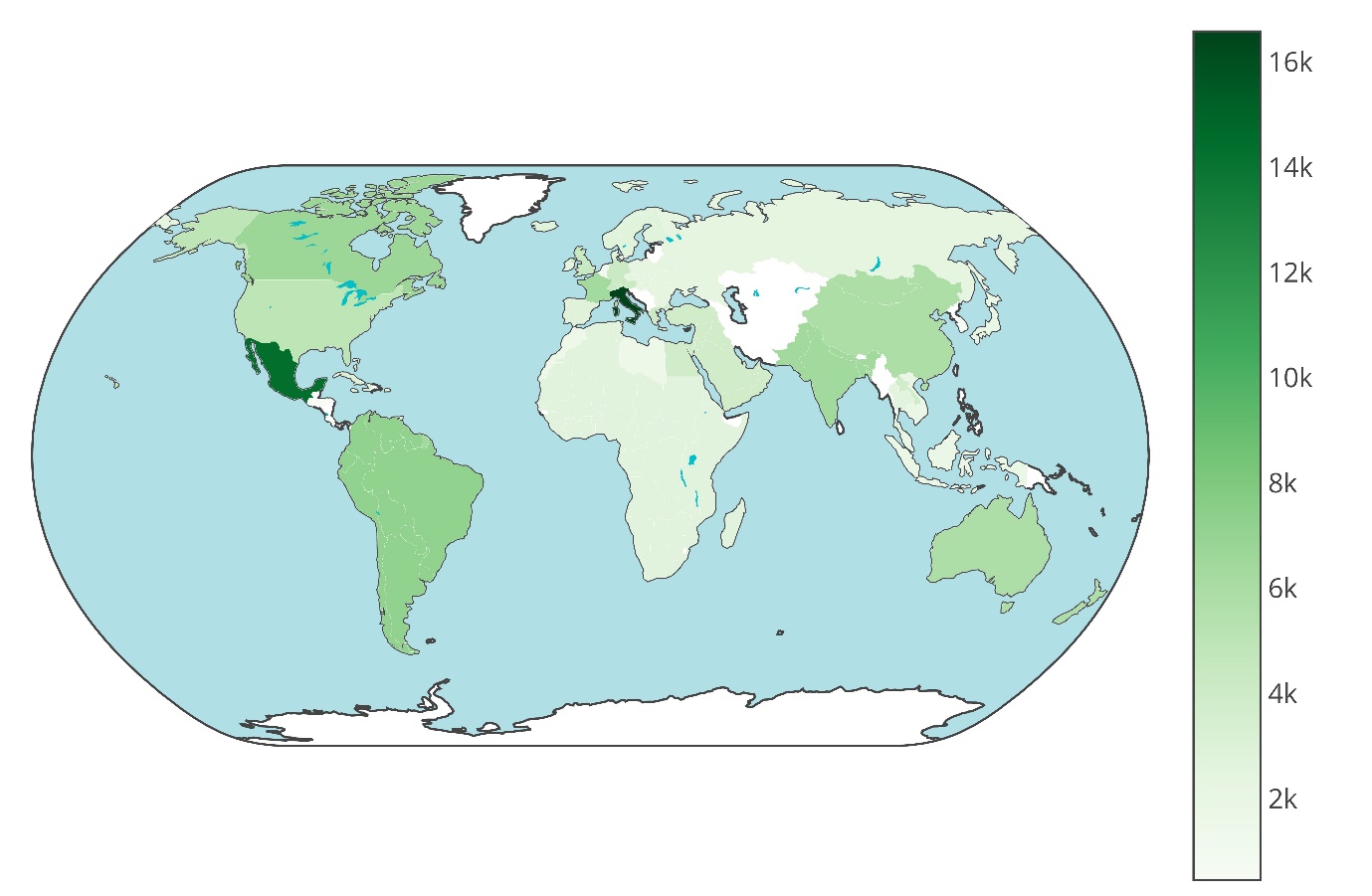


**Supplementary Figure S3**: Number of recipes across cuisines. The color gradient represents the number of recipes from each cuisine.


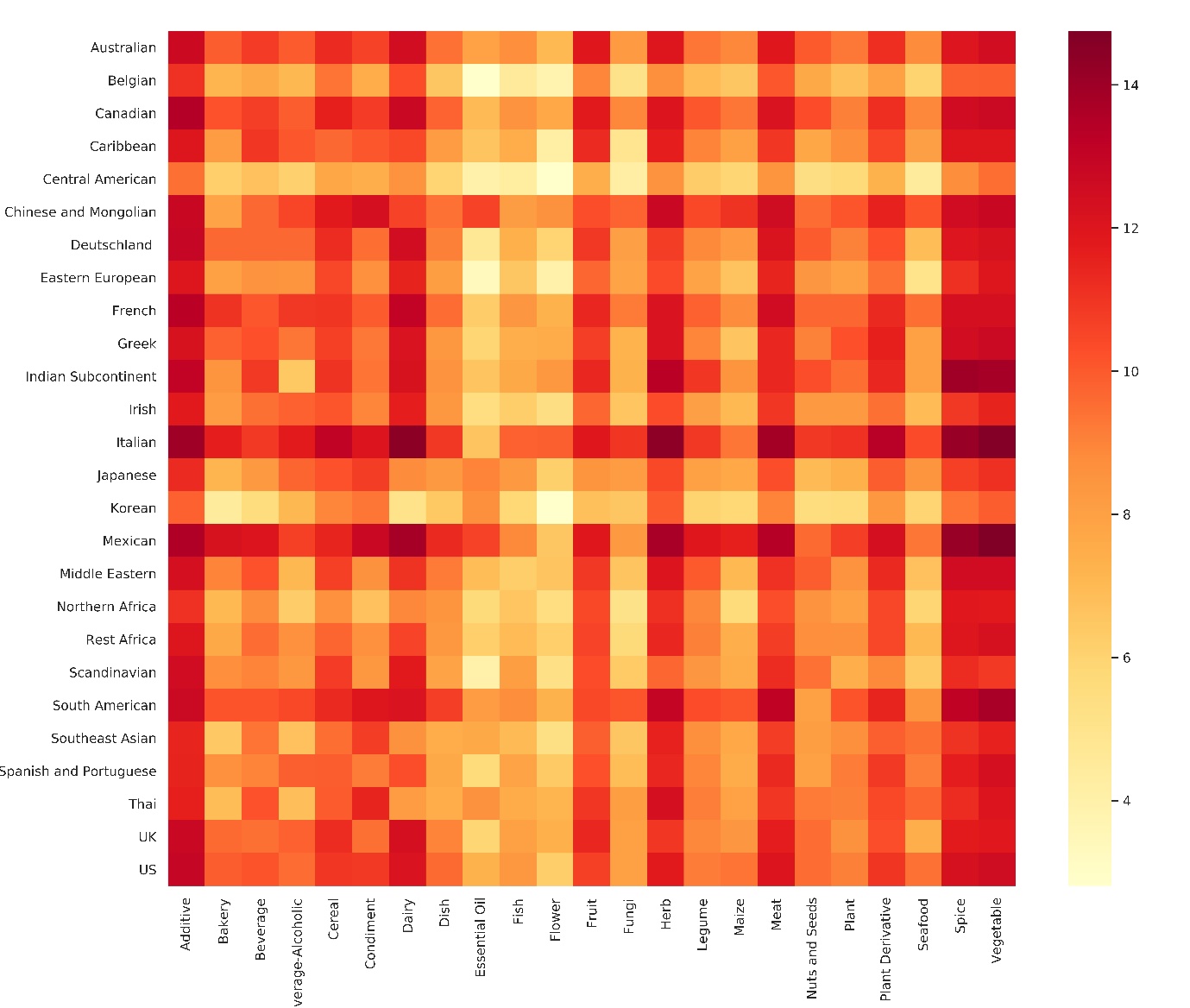


**Supplementary Figure S4**: Frequency of ingredients of each category in a cuisine (at region-level). This heatmap shows statistics of ingredient categories that dominate the cuisine versus those which are used relatively less frequently.


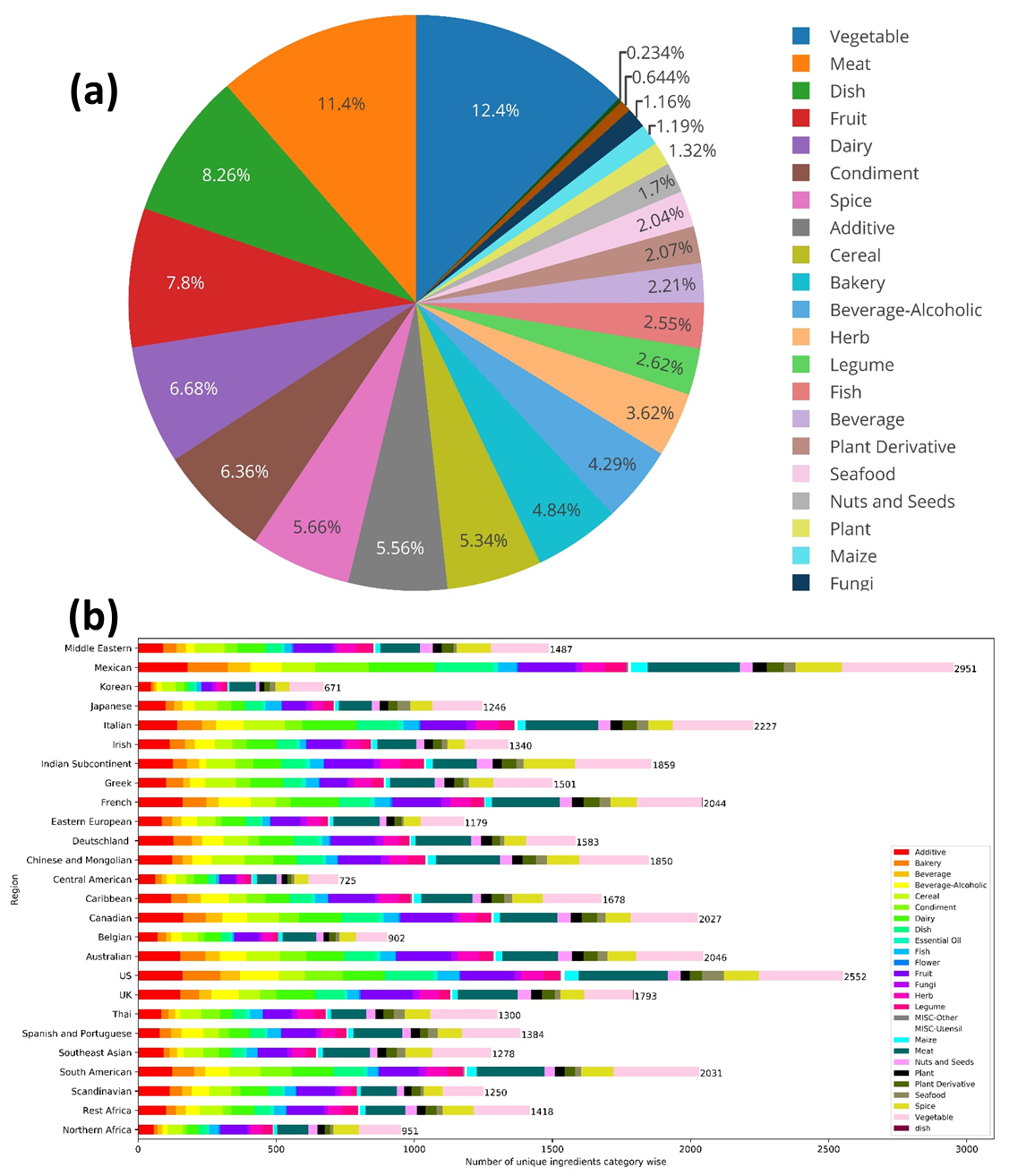


**Supplementary Figure S5**: (a) The pie chart shows the representation of each category of ingredients in the basket of ingredients that are used across the world cuisine. (b) The bar chart shows the statistics for cuisines at the region-level.


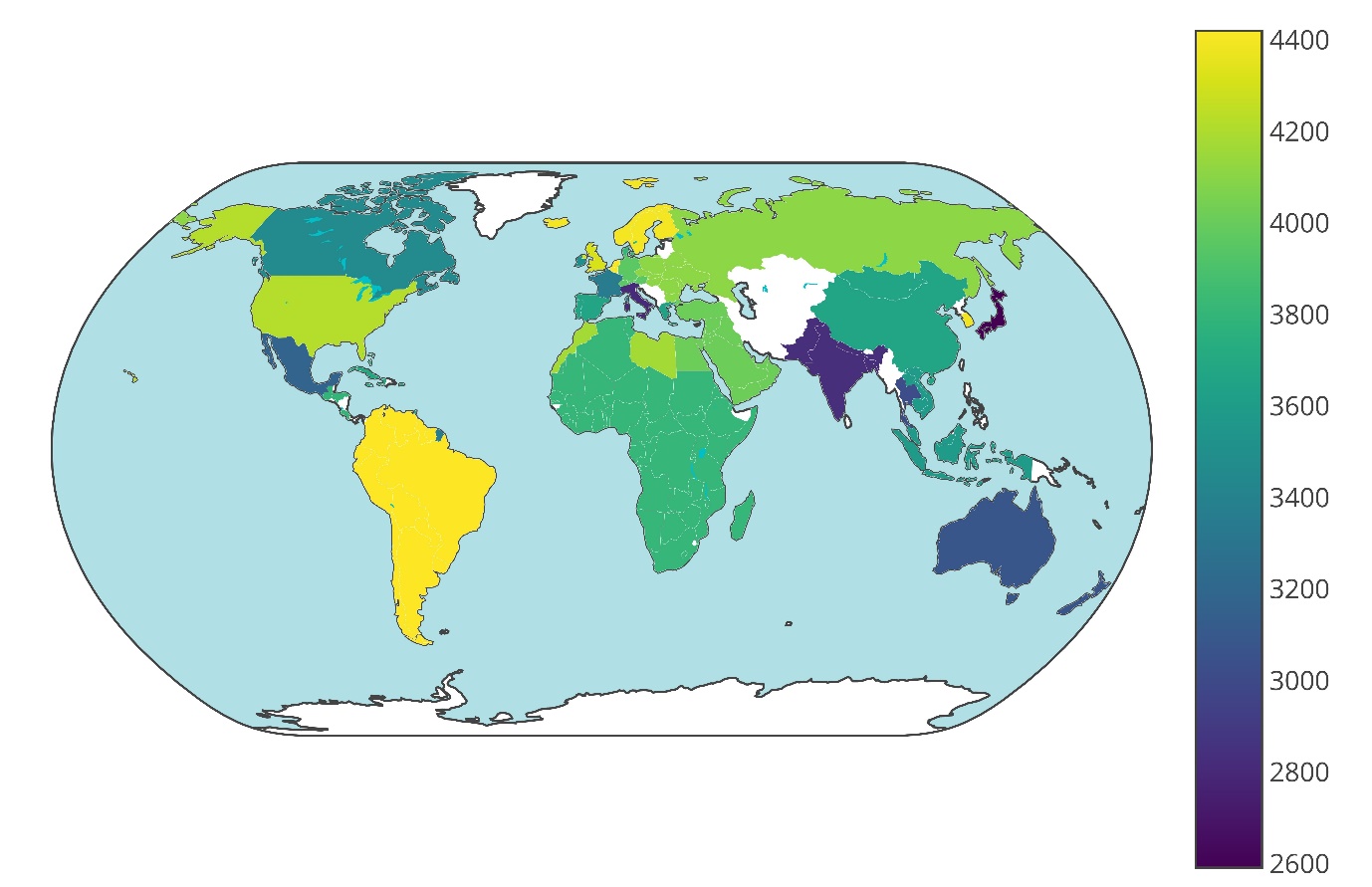


**Supplementary Figure S6**: Average calories across cuisines at the region level.
